# Comparative Phylogeography of Ethiopian anurans: impact of the Great Rift Valley and Pleistocene climate change

**DOI:** 10.1186/s12862-016-0774-1

**Published:** 2016-10-10

**Authors:** Xenia Freilich, José D. Anadón, Jolanta Bukala, Ordaliza Calderon, Ronveer Chakraborty, Stéphane Boissinot, Evolutionary Genetics - Class of 2013, Dina Calderon, Anastasia Kanellopoulos, Ewelina Knap, Paul Marinos, Maryam Mudasir, Stephen Pirpinas, Ryan Rengifo, Jayson Slovak, Alyssa Stauber, Edwin Tirado, Ivonne Uquilas, Michelle Velasquez, Elizabeth Vera, Anna Wilga

**Affiliations:** 1 Department of Biology, Queens College, City University of New York, Flushing, NY USA; 2 Ecology, Evolution and Behavior Program, Graduate Center, City University of New York, New York, NY USA; 3 New York University Abu Dhabi, P.O. Box 129188, Abu Dhabi, United Arab Emirates

**Keywords:** East Africa, Ethiopia, Great Rift Valley, Phylogeography, Anura, *Tomopterna*, *Amietia*, *Leptopelis*, *Ptychadena*

## Abstract

**Background:**

The Ethiopian highlands are a biodiversity hotspot, split by the Great Rift Valley into two distinct systems of plateaus and mountains. The Rift Valley is currently hot and dry and acts as a barrier to gene flow for highland-adapted species. It is however unlikely that the conditions in the Rift were inhospitable to highland species during the entire Pleistocene. To assess the significance of the Ethiopian Rift as a biogeographic barrier as well as the impact Pleistocene climatic changes have had on the evolution of Ethiopian organisms, we performed phylogeographic analyses and developed present and past niche models on seven anuran species with different elevational and ecological preferences.

**Results:**

We found that highland species on the east and the west sides of the Rift are genetically differentiated and have not experienced any detectable gene flow for at least 0.4 my. In contrast, species found at elevations lower than 2500 m do not show any population structure. We also determined that highland species have lower effective population sizes than lowland species, which have experienced a large, yet gradual, demographic expansion, starting approximately half a million year ago.

**Conclusions:**

The pattern we report here is consistent with the increasingly warmer and drier conditions of the Pleistocene in East Africa, which resulted in the expansion of savanna, the fragmentation of forests and the shrinking of highland habitats. Climatic niche models indicated that the Rift is currently non suitable for most of the studied species, but it could have been a more permeable barrier during the Last Glacial Maximum. However, considering the strong genetic structure of highland species, we hypothesize that the barrier mechanisms at the Rift are not only climatic but also topographical.

**Electronic supplementary material:**

The online version of this article (doi:10.1186/s12862-016-0774-1) contains supplementary material, which is available to authorized users.

## Background

The Ethiopian highlands constitute the largest continuous mountain system in Africa. These highlands are a biodiversity hotspot [1], particularly for amphibians as ~40 % of the species found in the highlands are endemic [2, 3]. The Ethiopian highlands are split by the Great Rift Valley (GRV) in two systems of plateaus and mountains: the Western highlands (or Abyssinian massif) and the Eastern highlands (or Harar massif). The GRV is the result of the splitting of the African plate into two, which began ~20 my ago [4, 5]. The climatic conditions on the highlands are relatively cool and humid while the GRV and the low-elevation areas surrounding the highlands are dry and hot, but this was not always the case. Since the late Miocene, the climate of East Africa has become increasingly arid and unstable [5, 6], with dry periods alternating with wet ones [6–10], coinciding with glaciations at northern latitudes [11]. For the Last Glacial Maximum (LGM, 23–18 ka before present) different global circulation models, such as those used in this work (see Methods), predict cooler and wetter conditions for East Africa, including the Ethiopian highlands ([12] but see [10]). These climatic cycles are expected to have promoted changes in the connectivity across the GRV and thus affected the possibility of gene flow between the Western and Eastern highlands. During drier periods the GRV could have acted as a barrier to dispersal for highland-adapted species as their distribution was being pushed to higher elevations. When the climate was wetter, however, highland species could have expanded their distribution toward low elevations and could have eventually dispersed across the GRV.

The effect the GRV had on population structure has been investigated in a handful of species, including charismatic taxa such as the Ethiopian wolf, the gelada baboon and the giant lobelia [13–15]. In all species examined, there is a clear genetic break between populations situated on the East and the West of the GRV, suggesting the Rift constitutes a major barrier to dispersal in these organisms. However, the east-west genetic break is recent in the Ethiopian wolf and probably followed the last de-glaciation event 15,000 years ago [13], whereas populations of the frogs *Xenopus clivii* and *X. largeni* have been isolated on each sides of the Rift for ~1–3.5 million years, with little or no migration [16]. At this point, the significance of the GRV as a biogeographic barrier has been examined in a very small number of species and further comparative studies are necessary to determine how the Rift Valley has affected dispersal in organisms with different ecological requirements.

We decided to examine the impact the GRV has had on the population structure of seven frog species with different ecology, behavior and habitat preference: *Amietia* sp*.*, *Leptopelis gramineus*, *Tomopterna kachowskii*, *Ptychadena cooperi*, *P. erlangeri* and two recently identified cryptic species, *P.* cf. *neumanni* 1 and *P.* cf. *neumanni* 2 [17]. The systematics of frogs belonging to the genus *Amietia* is still debated. Although populations from Ethiopia have been assigned to the species *Amietia angolensis* [2, 3], it is highly likely that this name recovers a species complex [18, 19] and that Ethiopian populations belong to a separate species. We refer to those frogs as *Amietia* sp. until their systematics has been resolved. *Amietia* sp. is relatively abundant on the Ethiopian plateaus, east and west of the Rift, and is exclusively found along large perennial streams [3]. Based on our observations, *Amietia* sp. is absent from the GRV and from the low elevation areas surrounding the Ethiopian highlands. *Leptopelis gramineus* is a fossorial and secretive species which is endemic to Ethiopia [3]. This species is commonly found on the plateaus but it can also reach high elevations, having been reported in the afro-alpine habitat. It is also found in the tropical humid forests of the Kaffa zone (southwest Ethiopia) and in the forests flanking the southern portion of the eastern highlands, only avoiding dry areas, such as the floor of the GRV. *Tomopterna kachowskii* is endemic to the horn of Africa and belongs to a genus commonly referred as sand frogs [20]. It has been collected at intermediate elevations on both side of the GRV and on the floor of the GRV, indicating it can tolerate dry conditions [20]. Frogs of this genus are known to survive dry conditions by digging burrows in sandy soils (hence their name) where they can aestivate for extended periods of time [21]. The four *Ptychadena* species analyzed here have a very low tolerance to dry conditions and are therefore absent from savannas and other arid environments. *Ptychadena* cf. *neumanni* 1 and 2 are typical grassland species that differ by their elevational range; *P.* cf*, neumanni* 1 is found exclusively below 2500 m while *P*. cf. *neumanni* 2 occurs between 2500 and 3100 m. *Ptychadena* cf. *neumanni* 1 is also found in clearings in the tropical humid forests of Kaffa. *Ptychadena erlangeri* is typically found in the moist evergreen forests of the Kaffa zone where it occurs in sympatry with *P.* cf. *neumanni* 1, although these two species differ in size and tend to occupy different micro-habitats [3, 17]. *Ptychadena cooperi* is the largest species of the genus analyzed here. It is found at elevations between 2400 and 3100 m and favors slow-running streams and large ponds. We performed a phylogeographic analysis of these seven taxa using a combination of mitochondrial and nuclear loci and we inferred the impact the GRV has had on their genetic structure. Results from phylogeographic analyses were complemented with the estimation of the changes in habitat suitability that occurred between the present time and the Last Glacial Maximum (LGM), as assessed by means of ecological niche modeling [22].

## Results

### Highland species: *Amietia* sp.*, Ptychadena cooperi* and *Ptychadena* cf. *neumanni* 2

*Ptychadena* cf. *neumanni 2*, *P. cooperi* and *Amietia* sp. were collected in the Ethiopian highlands, on both sides of the GRV, at elevations between 2364 and 3087 m asl. Although these three species are found in the same elevational range, they differ considerably in their use of microhabitats. *Ptychadena* cf. *neumanni 2* was collected in flooded grasslands, while *Amietia* sp*.* was found exclusively in large perennial streams. *Ptychadena cooperi* was always found near water, usually large ponds and slow running streams and rarely in flooded grasslands and never in the large streams favored by *Amietia* sp.

**Fig. 1:**
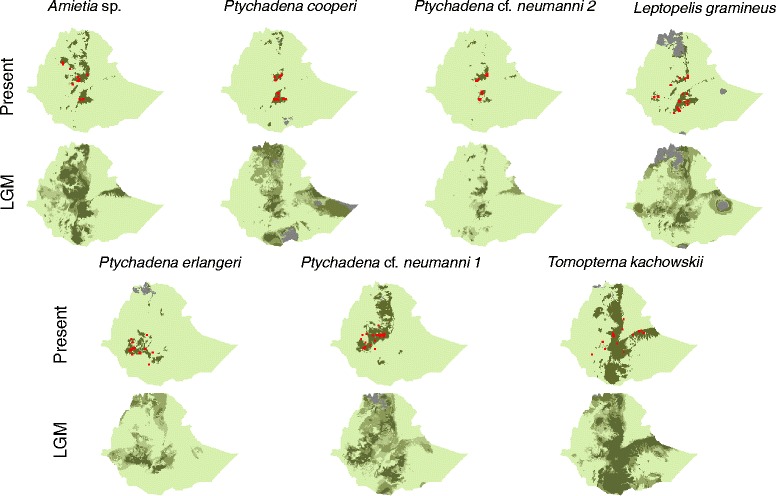
Predicted distribution of the seven species for the present time and the LGM as assessed from ecological niche modeling. For the LGM, the color gradient indicates for each pixel the number of scenarios (out of three) that predict that pixel to be part of the distribution range. The red squares indicate occurrence of each species. In grey are those pixels where the predictions under the three scenarios are uncertain due to non-analogous climatic conditions (see Methods for details)

Ecological Niche Models (ENM) for these three species showed high AUC values, particularly for *Ptychadena* cf. *neumanni* 2 and *P. cooperi* (Table 1). Predicted distribution ranges for the present time were similar among the three species and indicate that current favorable conditions are limited to the highlands (Fig. 1). ENMs indicated that the GRV is not a suitable area for any of these species at the present time. Projection of the ENMs for the LGM suggested that populations of *P. cooperi* and *Amietia* sp. east and west of the GRV could have been connected, whereas the GRV was probably not climatically suitable for *P.* cf. *neumanni* 2 (Fig. 1).

**Table 1:** Number of occurrence data (2.5 min pixels) employed for the ENM and AUC evaluation metric of the ENM for the different species

	Presence	AUC
*Amietia* sp*.*	13	0.875
*P. cooperi*	18	0.965
*P.* cf. *neumanni* 2	15	0.968
*L. gramineus*	24	0.913
*P. erlangeri*	17	0.877
*P.* cf. *neumanni* 1	24	0.869
*T. kachowskii*	16	0.699

**Fig. 2:**
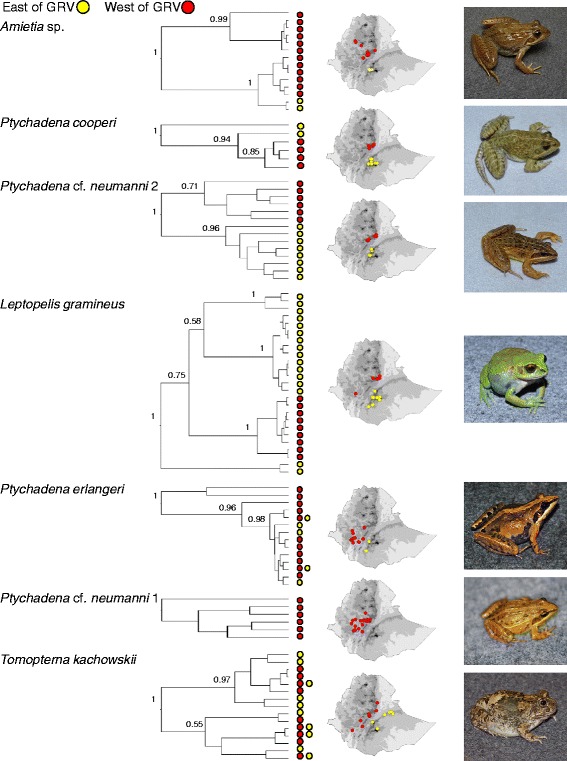
Mitochondrial phylogeny of Ethiopian anurans constructed using BEAST, maps of Ethiopia showing collecting localities and pictures of the organisms. The numbers at the nodes correspond to posterior probability values. Trees are not to scale

**Fig. 3:**
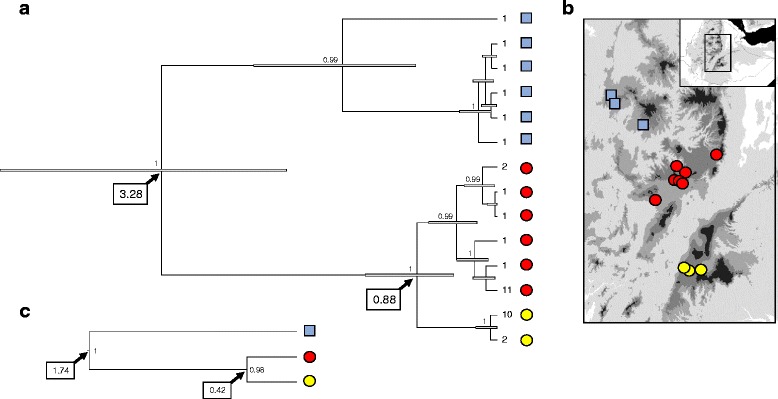
**a** Mitochondrial phylogeny of *Amietia* sp. constructed using BEAST. The numbers at the nodes correspond to posterior probability values. The boxed number corresponds to the age of the nodes. **b** Map showing the origin of the samples. C. Population phylogeny reconstructed using *BEAST. The boxed numbers correspond to the nodes age calibrated using the ND2 mutation rate (0.957 %)

All phylogenetic analyses of the mitochondrial sequences revealed similar patterns between the three species, with a clear clustering of mitochondrial haplotypes from the same side of the GRV (Fig. 2; only the BEAST analyses are shown). In *P*. cf. *neumanni 2*, the 14 mitochondrial haplotypes cluster into two well supported clades corresponding perfectly to the geographical origin of the samples, one clade containing all the frogs collected in the west and the other one the frogs from the east of the GRV (Fig. 2). In *Amietia* sp*.*, we recovered a similar east-west split but we identified a divergent clade corresponding to populations located north of the Blue Nile valley (Fig. 3). In *P. cooperi*, we recovered only 6 haplotypes, 4 of which are unique to the west (Fig. 2). The western haplotypes form a monophyletic clade, which is nested within a paraphyletic eastern group. The two eastern haplotypes have distinct geographic distribution, one being widespread on the plateau while the most divergent one is found at high elevation in the Bale massif. The average divergence between the western and the eastern populations are similar among species with values of 1.3, 1.8 and 2.0 % in *Amietia* sp., *P*. cf. *neumanni 2* and *P. cooperi*, respectively. The AMOVAs based on the mitochondrial data confirm the east-west split since a significant amount of the variance was explained by among group variation, the groups being defined as the populations east and west of the GRV (Table 2).

**Table 2:** Mitochondrial and nuclear DNA hierarchical analysis of molecular variance (AMOVA) and coefficient of differentiation between east and west populations. Groups were defined a priori and contain populations east and west of the GRV

Source of variation	Mitochondrial DNA		Nuclear DNA		*F*_*st*_ east-west
	% of variation	*P*-value	% of variation	*P*-value	All nuclear loci
*Amietia* sp*.*					
Among groups	84.99	0.0127	69.89	0.0098	0.644
Within groups	10.84	0.0039	6.79	0.0019	
Within populations	4.16	0.0000	23.32	0.0000	
*Ptychadena cooperi*					
Among groups	47.11	0.0362	61.68	0.0049	0.634
Within groups	50.53	0.0000	7.21	0.0000	
Within populations	2.36	0.0000	31.12	0.0000	
*Ptychadena* cf. *neumanni* 2					
Among groups	76.57	0.0039	38.31	0.0088	0.410
Within groups	10.54	0.0000	7.93	0.0000	
Within populations	12.89	0.0000	53.76	0.0000	
*Leptopelis gramineus*					
Among groups	52.15	0.0039	47.76	0.0049	0.504
Within groups	34.01	0.0000	12.55	0.0000	
Within populations	13.84	0.0000	39.68	0.0000	
*Ptychadena erlangeri*					
Among groups	−13.33	0.4946	4.13	0.0958	0.061
Within groups	23.19	0.1906	3.86	0.0498	
Within populations	90.14	0.1740	92.01	0.0020	
*Tomopterna kachowskii*					
Among groups	−13.97	0.9990	−0.18	0.4624	0.024
Within groups	46.56	0.0000	1.08	0.3617	
Within populations	67.41	0.0000	99.1	0.3724	

The amount of nuclear variation differs substantially among species and tends to be higher in *P.* cf. *neumanni 2* (38 SNPs in total) than in *P. cooperi* (17 SNPs) and *Amietia* sp. (17 SNPs), which exhibit similar level of variation (Table 3). Despite an overall low level of variation, the nuclear datasets contain significant phylogeographic information, including a number of SNPs that are diagnostic of the populations east and west of the GRV. For instance, a SNP in *Rag-1* is diagnostic of the western populations of *P. cooperi* and two SNPs in *BDNF* and *Tyr* are diagnostic of the western population in *Amietia* sp.. Consistent with the presence of population-specific SNPs, the *Structure* and *Structurama* analyses strongly support the existence of two gene pools separated by the GRV in all three species (Table 4). The genetic differentiation between east and west is also confirmed by the AMOVA analysis (Table 1), which estimated that a large and significant fraction of the genetic variance is found among groups. Finally, the *F*_*st*_ values calculated on a concatenated nuclear dataset are high and significant for all species (Table 2). Together, the mitochondrial and nuclear data strongly support the presence of two distinct geographic populations separated by the GRV. The presence of a third population north of the Nile in *Amietia* sp. was not recovered by the *Structurama* and *Structure* analyses, probably because of the small number of loci used and the low nuclear variation recovered in this species. However, the high and significant *F*_*st*_ values (Table 5) suggest that the population north of the Nile is indeed genetically differentiated from the other two (Fig. 3).

**Table 3:** Summary statistics of diversity and differentiation for mitochondrial and nuclear genes

*Ametia* sp*.*												
	ND2		Rag-1		Tyr		BDNF					
	West	East	West	East	West	East	West	East				
N	12	19	14	28	22	32	24	26				
S	13	24	2	2	3	3	1	1				
Π	0.239	0.399	0.085	0.105	0.108	0.074	0.07	0.026				
θ	0.447	0.715	0.08	0.065	0.152	0.138	0.048	0.047				
Tajima’s D	−0.213	−1.723	0.179	1.268	−0.749	−1.079	−0.741	0.363				
Fu’s F	1.885	0.297	1.475	0.987	−0.271	−1.225	−0.381	0.394				
*F*_*st*_	0.761		0.449		0.646		0.176					
*Ptychadena cooperi*												
	COI		CXCR4		NCX1		Rag-1		Tyr			
	West	East	West	East	West	East	West	East	West	East		
N	21	10	60	26	62	22	62	16	62	24		
S	3	15	4	2	4	1	3	1	2	5		
Π	0.182	2.518	0.143	0.065	0.033	0.016	0.031	0.049	0.158	0.464		
θ	0.339	1.907	0.192	0.117	0.150	0.048	0.152	0.119	0.129	0.405		
Tajima’s D	−1.217	1.475	−0.558	−0.960	−1.684	−1.162	−1.576	−1.162	0.381	0.427		
Fu’s F	−1.770	9.345	−1.176	−1.046	−2.975	−0.957	−3.984	−0.700	0.591	−1.672		
*F*_*st*_	0.478		0.712		0.001		0.942		0.275			
*Ptychadena* cf.*neumanni* 2												
	COI		CXCR4		NCX1		Rag-1		Tyr			
	West	East	West	East	West	East	West	East	West	East		
N	36	24	80	62	76	54	64	60	78	62		
S	8	9	3	7	3	5	7	14	3	10		
Π	0.307	0.447	0.032	0.111	0.064	0.667	0.196	0.865	0.320	1.006		
θ	0.694	0.867	0.135	0.381	0.102	0.193	0.327	0.629	0.216	0.710		
Tajima’s D	−1.625	−1.588	−1.416	−1.859	−0.708	−1.533	−1.014	1.103	0.910	1.150		
Fu’s F	−1.896	−3.562	0.021	−4.412	−1.185	−5.661	−0.230	1.386	−0.011	−1.157		
*F*_*st*_	0.793		0.039		0.016		0.274		0.555			
*Leptopelis gramineus*												
	COI		Rag-2		NCX1		Rag-1		Tyr		BDNF	
	West	East	West	East	West	East	West	East	West	East	West	East
N	9	16	18	50	18	48	18	50	18	40	18	50
S	18	68	4	23	1	10	8	19	7	15	3	5
Π	1.275	4.118	0.252	0.463	0.02	0.269	0.279	0.361	0.353	0.511	0.149	0.121
θ	1.299	4.195	0.205	1.019	0.053	0.407	0.333	0.605	0.458	0.851	0.19	0.279
Tajima’s D (East = Arsi)	−0.267	−0.585	0.676	−1.232	−1.165	−1.524*	−0.565	−1.209	−1.042	−0.278	−0.591	−0.964
Fu’s F (East = Arsi)	−2.843	−1.987	−0.362	−4.967*	−0.794	−2.113	0.444	−4.455*	−1.584	−5.203*	−0.964	−2.707*
*F*_*st*_	0.537		0.125		0.077		0.561		0.353		0.008	
*Ptychadena erlangeri*												
	COI		CXCR4		NCX1		Rag-1		Tyr			
	West	East	West	East	West	East	West	East	West	East		
N	28	8	68	20	60	18	68	16	70	16		
S	40	7	15	5	7	0	15	11	29	11		
Π	2.024	0.503	0.366	0.269	0.104	NA	0.493	0.625	1.261	0.743		
θ	3.790	0.690	0.794	0.38	0.266	NA	0.690	0.695	2.048	0.730		
Tajima’s D	−1.740	−1.309	−1.634	−0.965	−1.561	NA	−0.832	−0.378	−1.221	0.068		
Fu’s F	−0.039	−2.317	−12.096	−2.94	−5.153	NA	−5.004	−1.501	−27.296	−1.120		
*F*_*st*_	0.029		0.055		0.032		0.029		0.109			
*Ptychadena* cf. *neumanni* 1												
	COI		CXCR4		NCX1		Rag-1		Tyr			
	West	East	West	East	West	East	West	East	West	East		
N	49		126		114		118		122			
S	8		9		5		12		20			
Π	0.322		0.253		0.121		0.264		0.859			
θ	0.645		0.372		0.166		0.575		1.348			
Tajima’s D	−1.371		−0.775		−0.567		−1.426		−1.024			
Fu’s F	1.073		−2.142		−2.409		−6.391		−12.296			
*Tomopterna kachowskii*												
	COI		SLC8A3		NCX1		Rag-1		BDNF			
	West	East	West	East	West	East	West	East	West	East		
N	21	15	50	30	44	30	46	28	42	30		
S	16	22	5	2	9	9	8	6	4	3		
Π	1.062	1.135	0.151	0.042	0.231	0.193	0.454	0.358	0.215	0.214		
Θ	0.884	1.401	0.299	0.109	0.448	0.287	0.462	0.325	0.204	0.168		
Tajima’s D	−1.257	−2.176 *	−1.2006	−1.2555	−1.3893	−1.0164	−0.053	0.2998	0.1256	0.6543		
Fu’s F	0.358	−0.697	−1.661	−1.021	−0.976	−1.142	−3.341	−1.995	0.003	1.002		
*F*_*st*_	−0.017		0.109		0.001		−0.028		−0.015			

*N* number of individuals, *S* number of SNPs, *Π* nucleotide diversity, *θ* Waterson’s estimator of diversity. Values of Tajima’s D and Fu’s F that are significantly different from 0 are indicated with *

**Table 4:** Structure and Structurama analysis based on nuclear loci

Species	K	Structure			Structurama
		lnP	Pr(K|X)	ΔK	
*Amietia* sp*.*	1	−296.98	0.00	NA	0.00
	2	−169.80	0.00	1292.20	0.99
	3	−171.84	0.00	12.22	0.01
	4	−153.88	0.99	15.80	0.00
	5	−172.26	0.00	2.67	0.00
	6	−182.58	0.00	NA	0.00
*Ptychadena cooperi*	1	−423.72	0.00	N/A	0.00
	2	−265.46	1.00	2725.83	1.00
	3	−256.50	0.00	10.76	0.00
	4	−255.52	0.00	6.82	0.00
	5	−264.68	0.00	0.83	0.00
	6	−277.18	0.00	N/A	0.00
*Ptychadena* cf. *neumanni* 2	1	−1471.56	0.00	N/A	0.00
	2	−1035.66	0.99	422.87	1.00
	3	−797.20	0.00	182.78	0.00
	4	−741.52	0.00	2.90	0.00
	5	−745.58	0.00	0.95	0.00
	6	−700.80	0.00	N/A	0.00
*Leptopelis gramineus*	1	−1446.66	0.00	NA	0.00
	2	−1072.24	0.00	514.90	1.00
	3	−978.90	0.00	2.69	0.00
	4	−1018.82	0.00	0.68	0.00
	5	−888.98	0.00	5.66	0.00
	6	−869.12	0.99	NA	0.00
*Ptychadena erlangeri*	1	−1303.78	1.00	N/A	1.00
	2	−1304.78	0.00	57.06	0.00
	3	−1438.96	0.00	5.60	0.00
	4	−1312.12	0.00	4.79	0.00
	5	−1346.86	0.00	22.13	0.00
	6	−1311.38	0.00	N/A	0.00
*Ptychadena* cf*. neumanni* 1	1	−1074.62	1.00	N/A	1.00
	2	−1034.38	0.00	62.15	0.00
	3	−1032.80	0.00	10.43	0.00
	4	−1033.88	0.00	0.70	0.00
	5	−1035.16	0.00	1.81	0.00
	6	−1035.86	0.00	N/A	0.00
*Tomopterna kachowskii*	1	−513.10	0.99	NA	0.99
	2	−529.50	0.00	13.76	0.01
	3	−628.72	0.00	0.70	0.00
	4	−665.20	0.00	0.34	0.00
	5	−719.50	0.00	2.65	0.00
	6	−632.14	0.00	NA	0.00

For Structure, the probability of each K given the data (Pr(K|X)) was calculated using the ad hoc method described by Pritchard et al. [39]. The optimal number of K was also determined using the ΔK method of Evanno et al. [40]. For the Structurama we present the posterior probability assuming a gamma hyper-prior G(2.5,0.5)

**Table 5:** Coefficient of differentiation (*F*_*st*_) among populations of *Leptopelis gramineus* and *Amietia sp.* based on all nuclear loci

*Amietia* sp*.*	East	North		
North	0.4385			
West	0.6436	0.7405		
*Leptopelis gramineus*	Arsi	Kasha	Kibre Mengist	All Eastern Populations
Kasha	0.2281			
Kibre Mengist	0.0825	0.1227		
West	0.5827	0.5321	0.5678	0.5037

The BEAST estimates of divergence time between the east and west mitochondrial lineages are similar among species and suggests a split ~0.8 to 1.0 my ago, in the mid-Pleistocene (Table 6). The *BEAST analyses, which take into account the coalescent of the mitochondrial and nuclear loci, produced similar estimates among species and support divergences in the Pleistocene, 0.39 to 0.50 my ago (Table 6). The BEAST estimates tend to be older than the *BEAST estimates but this is expected since divergence between alleles pre-dates population divergence. Finally, the East-west split estimated with G-PHoCS (Table 7), using only the nuclear genes and a different mutation rate than *BEAST, yielded close estimates from 0.34 to 0.53 my. The estimates of divergence of the northern populations of *Amietia* sp. were different across methods, with a BEAST estimate at 3.28 my, a *BEAST estimate at 1.74 my and a G-PHoCS estimate at 0.65 my. This is likely caused by the discordance between the large mitochondrial divergence and the low level of differentiation observed at nuclear loci.

**Table 6:** Time of divergence

		BEAST	*BEAST
*Amietia* sp*.*	TMRCA - East	0.12 (0.03–0.26)	_
	TMRCA - West	0.39 (0.19–0.64)	_
	TMRCA - North	1.45 (0.59–2.23)	_
	Divergence (East, West)	0.88 (0.48–1.45)	0.42 (0.09–3.10)
	Divergence North (East, West)	3.28 (1.75–4.98)	1.74 (0.58–5.12)
*Ptychadena cooperi*	TMRCA - West	0.29 (0.09–0.62)	_
	Divergence (East, West)	0.82 (0.28–1.54)	0.50 (0.14–1.06)
	Divergence Bale (East, West)	2.84 (0.85–4.60)	_
*Ptychadena* cf*. neumanni* 2	TMRCA - East	0.61 (0.11–2.02)	_
	TMRCA - West	0.63 (0.12–1.98)	_
	Divergence (East, West)	1.05 (0.54–1.80)	0.39 (0.05–1.92)
*Leptopelis gramineus*	TMRCA - Arsi	0.67 (0.36–1.08)	_
	TMRCA - West	1.34 (0.76–2.10)	_
	TMRCA - Kibre Mengist	0.99 (0.47–1.71)	_
	TMRCA - Bale	0.37 (0.10–0.79)	_
	Divergence - node 1	5.47 (3.66–7.83)	2.00 (1.02–3.34)
	Divergence - node 2	4.26 (3.06–5.85)	1.21 (0.70–1.94)
	Divergence - node 3	3.62 (2.38–5.06)	0.95 (0.52–1.56)
*Ptychadena erlangeri*	TMRCA	5.13 (3.96–6.38)	_
	TMRCA main clade	0.51 (0.24–0.78)	_
*Ptychadena* cf. *neumanni* 1	TMRCA	0.74 (0.29–1.20)	_
*Tomopterna kachowskii*	TMRCA	0.86 (0.45–0.14)	_

Ages are in million years and the numbers in parentheses correspond to the 95 % highest posterior density. Node numbers as on Fig. 5

**Table 7:** Coalescent based estimates for population parameters. The estimates of divergence time (τ in years), population size (θ in individuals) and migration rate (m) are shown

	Parameters	Mean (95 % CI lower - 95 % CI upper)
*Amietia* sp*.*		
	θ west	38,710 (12,186–70,967)
	θ east	77,957 (26,881–138,172)
	θ north	53,405 (11,469–104,659)
	θ west-east	55,197 (5734–115,949)
	θ root	51,612 (2508–111,469)
	τ west-east	417,491 (147,096–708,243)
	τ root	652,043 (272,401–1,089,605)
	m west = > east	0.0022 (0.0000–0.0114)
	m east = > west	0.0010 (0.0000–0.0058)
	m west = > north	0.0006 (0.0000–0.0032)
	m north = > west	0.0008 (0.0000–0.0051)
*Ptychadena cooperi*		
	θ west	38,631 (15,890–69,870)
	θ east	65,707 (24,059–115,415)
	θ west-east	63,346 (20,199–111,511)
	τ west-east	528,861 (277,923–906,446)
	m east = > west	0.0001 (0–0.0008)
	m west = > east	0.0001 (0–0.0008)
*Ptychadena* cf*. neumanni* 2		
	θ west	27,212 (10,519–46,106)
	θ east	149,4546 (83,936–223,559)
	θ west-east	198,253 (82,233–275,627)
	τ west-east	339,231 (134,523–579,477)
	m east = > west	0.0001 (0–0.0009)
	m west = > east	0.0001 (0–0.0016)
*Leptopelis gramineus*		
	θ Arsi	1,017,025 (476,882–1,617,025)
	θ Kibre Mengist	576,165 (282,975–913,262)
	θ Kasha	831,720 (271,685–1,482,079)
	θ West	517,383 (265,771–808,781)
	θ Arsi-Kibre Mengist	797,670 (89,964–1,652,151)
	θ Arsi-Kibre Mengist-Kasha	1,022,939 (232,079–1,899,283)
	θ root	554,839 (252,151–901,971)
	τ Arsi-Kibre Mengist	666,953 (180,645–1,129,749)
	τ (Arsi-Kibre Mengist)-Kasha	1,050,896 (539,068–1,628,673)
	τ root	2,097,204 (1,353,405–2,864,516)
	m west = > east	0.0001 (0.0000–0.0007)
	m east = > west	0.0001 (0.0000–0.0007)

**Fig. 4:**
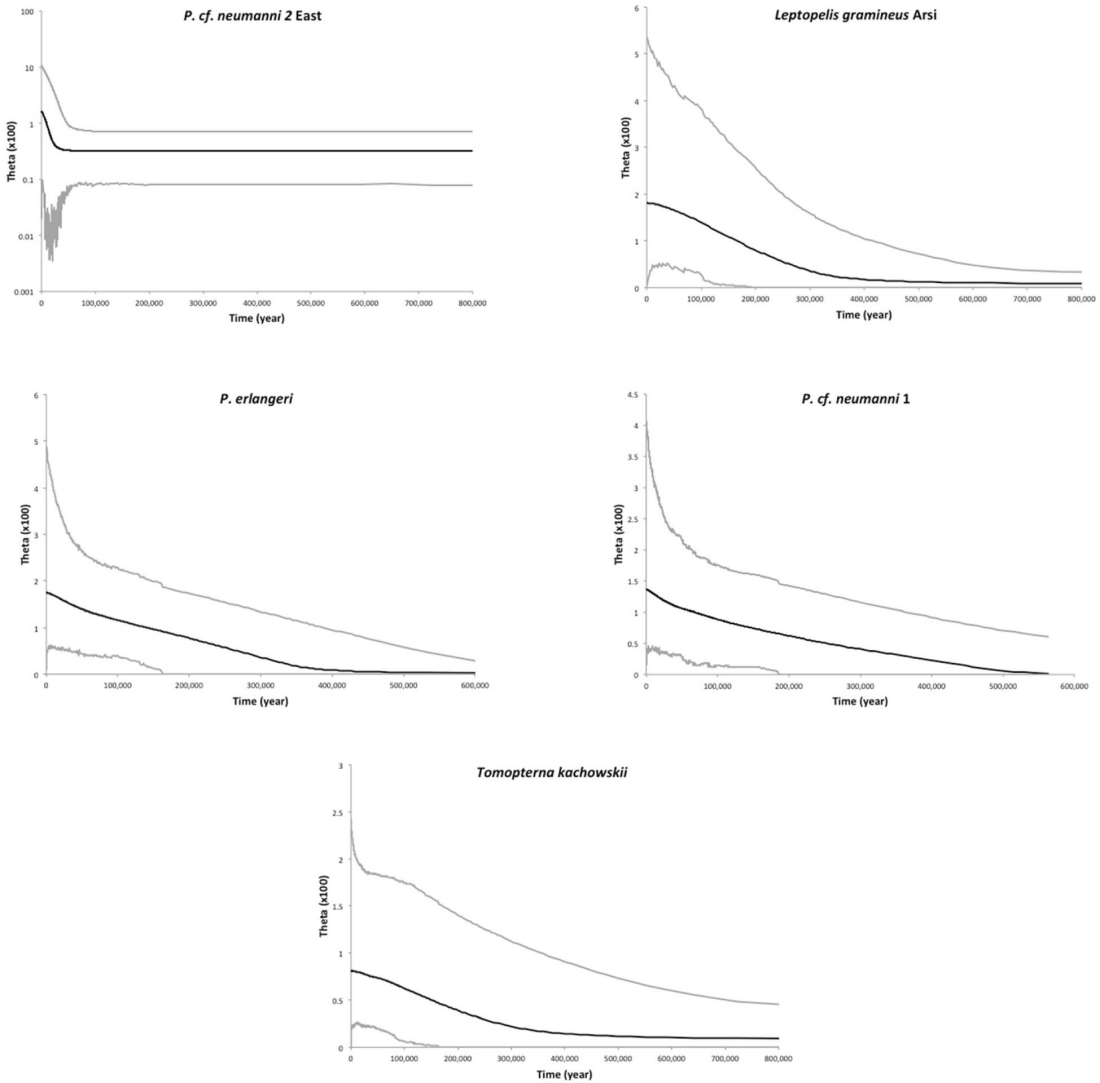
Demographic trajectories estimated by Extended Bayesian Skyline Plot (EBSP). The median population size is shown with a black line and the 95 % credible intervals are shown with grey lines

The estimates of effective population size differ among species (Table 7), which is consistent with the substantial differences in genetic diversity at all loci (Table 3). The estimates of population size for *P. cooperi* and *Amietia* sp. are similar but in both species the population sizes on the east (~66,000 and 78,000 for *P. cooperi* and *Amietia* sp.) are substantially higher than on the west (~38,000). The same pattern is observed in *P*. cf. *neumanni 2* but the difference is much larger since the estimate of population size in the east is five times larger than the western one (149,455 *vs* 27,212). Migration rates estimated by G-PhoCS are extremely low in all three species with 95 % credible intervals containing 0 for both east-to-west and west-to-east estimates suggesting that the eastern and western populations have not exchanged migrants at a detectable level since their split (Table 7). The EBSP analysis (Fig. 4) performed on the western population of *P*. cf. *neumanni 2* suggests that this population experienced a rapid 10-fold increase in population size in the last 20,000 years. The EBSPs performed on the western population of *P.* cf. *neumanni* 2, *Amietia* sp. and *P. cooperi* are all indicative of a stable demography (not shown). However, the low amount of genetic variation in these populations is probably insufficient to accurately reconstruct the demographic history of these species and will require the acquisition of data from a large number of fast-evolving nuclear loci.

### A fossorial frog from the grasslands and the forests: *Leptopelis gramineus*

**Fig. 5:**
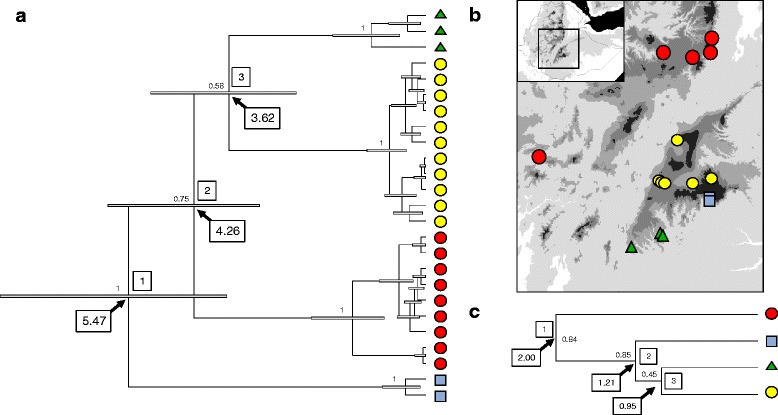
**a** Mitochondrial phylogeny of *L. gramineus* constructed using BEAST. The numbers at the nodes correspond to posterior probability values. The boxed number corresponds to the age of the nodes. **b** Map showing the origin of the samples. **c** Population phylogeny reconstructed using *BEAST. The boxed numbers correspond to the nodes age calibrated using the two mutation rates

Thirty-four specimens of *Leptopelis gramineus* were collected from 17 localities, 5 on the western side of the GRV and 12 on the eastern side (Fig. 5). The sampling sites on the east include 6 grassland localities ranging from 2400 to 3000 m asl and 6 localities in the tropical humid forest flanking the southern side of the eastern highlands, at elevations ~2100 to ~2400 m. Five of the western localities were in highland grasslands between ~2600 and ~2800 m asl whereas a single locality 30 km south west of Jimma was in a humid tropical forest at an elevation of ~2100 m. ENMs for *L. gramineus* showed good prediction ability (Table 1) and indicated that current favorable climatic conditions are limited to the highlands and to the humid forests of the south (Fig. 1). ENMs indicate that while the GRV is currently not favorable for the species, it might have been suitable during the LGM (Fig. 1).

All phylogenetic reconstructions based on the *COI* gene produced identical topologies indicative of four well-supported and highly divergent mitochondrial lineages (Fig. 2 and 5), with defined geographic distributions. The average distance between these clades is high, ranging from 6.4 to 8.0 %. One of the clades includes all haplotypes from the west side of the GRV whereas three clades are found in the east: a clade found on the Arsi plateau and at high elevations in the Bale massif (referred as the Arsi clade on Table 4), a clade found exclusively near Kasha in the Harenna forest (Kasha clade) and a clade found in the forest of the southernmost portion of the eastern massif, near the town of Kibre Mengist (Kibre Mengist clade). Interestingly the western clade is nested within the three eastern clades, which do not form a monophyletic group. Consistent with a clear east-west break and with the presence of divergent mitochondrial lineages in the east, the AMOVA reveals that 52 % of the mitochondrial variation is explained by differences between the east and the west of the GRV and 34 % by differences among populations within groups (Table 2).

The level of nuclear variation in *L. gramineus* is comparatively higher than in other species with a total of 94 SNPs. Although the number of SNPs is large, a single one (in the *Rag-1* gene) separates the populations west and east of the GRV. The *Structure* analysis supports the presence of two populations, separated by the GRV, by the delta-K criterion but six populations by the Pr(K|X) criterion. The Pr(K|X) criterion also separates the individuals of the western side of the GRV but does not reveal any biologically meaningful structure in the samples from the east. Consistent with the *Structure* analysis, *Structurama* recovers the same east-west split (Table 4). Interestingly, the clustering approaches did not recover any genetic structure in the east, although the mitochondrial analysis suggested the presence of three distinct populations. The AMOVA performed on the 5 nuclear genes confirmed the validity of the east-west break and the lack of strong structure within each group, as only 12.55 % of the variation is explained by variation among populations (Table 2). *F*_*st*_ (Table 5) calculated between the western population and each of the eastern populations yielded high and significant values (from 0.53 to 0.58) whereas *F*_*st*_ among eastern populations defined by their mitochondrial lineage yielded much lower, yet significant, values (from 0.08 to 0.23). Taken together, the analyses of the mitochondrial and nuclear datasets strongly indicate a clear genetic break between the eastern and western populations of *L. gramineus* as well as additional genetic structure between the eastern populations from the plateau and the populations from the tropical forests.

BEAST estimated that the four mitochondrial lineages diverged in the Pliocene, between 5.5 and 3.6 my (Fig. 5 and Table 6). The species tree approach yielded a different topology than the mitochondrial gene trees, the western population being the most divergent one and the three eastern populations forming a monophyletic group, as well as more recent divergence time (Fig. 5). *BEAST estimates the split between the western and the eastern populations in the early Pleistocene (2.0 my; Table 6). The divergence between the three eastern populations is dated at ~1.2 to 0.9 my. The G-PHoCS estimates of divergence are similar to the one obtained with *BEAST with an east-west divergence dated ~2.1 my in the early Pleistocene (Table 6).

The G-PHoCS estimates of effective population size are consistent across priors and relatively similar among populations, ranging from ~500,000 to ~1,000,000 individuals (Table 7). However, an EBSP analysis of the Arsi population suggests a 10-fold demographic expansion starting ~ 400,000 year ago (note that the sample size for the other populations was too low for EBSP analysis). A population expansion for Arsi is also supported by Tajima’s D and Fu’s F, which yielded negative values for all loci (mitochondrial and nuclear), although few values were significant. The migration rates estimated by G-PHoCS between the east and west populations are not significantly different from 0, indicating that the Western and Eastern populations have apparently not exchanged migrants at a detectable level for a very long time (Table 7).

### Lower elevation species: *Ptychadena erlangeri*, *P.* cf. *neumanni 1* and *Tomopterna kachowskii*

*Ptychadena erlangeri* was collected in forest clearings, in the southwest and in two localities east of the GRV. The distribution of *P*. cf. *neumanni 1* is limited to the west of the GRV where it occupies grassland habitats under 2500 m but its distribution extends to the forests of the southwest where it coexists with *P. erlangeri. Tomopterna kachowskii* were collected across a large range of elevations (from ~1300 to ~2600 m above sea level) and habitats (from dry lowland savanna to mid-elevation grasslands) on both side of the GRV.

ENMs for these species indicated distinct distribution ranges for the present time and different range dynamics since the LGM (Fig. 1). Evaluation metrics for these models showed good prediction ability for *P. erlangeri* and *P.* cf. *neumanni 1* but moderate for *T. kachowskii* (Table 1). The predicted distribution for *P. erlangeri* includes the forests of Kaffa as well as the forests that are flanking the southern side of the eastern highlands. Interestingly the model predicts the presence of a corridor of favorable habitat crossing the GRV, south of Awassa and north of Lake Abaya, suggesting a possible connection between populations east and west of the Rift (Fig. 1). According to the projection of ENMs, this species could have had a similar but somewhat more widespread distribution during the LGM than in the present time. ENMs for *P.* cf. *neumanni 1* suggest that favorable habitat for this species currently exists almost exclusively on the west side of the GRV and includes the entire western massif, excluding the highest elevation mountains. It also extends south, reaching the northern portion of the tropical forest of Kaffa. For the LGM, ENMs indicate that there could have been suitable areas for *P*. cf. *neumanni 1* on the Eastern Highlands and that the Western and Eastern highlands could have been connected by suitable habitats*.* Consistent with our observations and what is known of the ecology of this species, ENMs predict that the distribution of *T. kachowskii* is continuous in the lowlands and extends on the flanks of the highlands, but does not include the highest elevation areas. A similar distribution pattern is predicted for the LGM.

Phylogenetic analyses of the *COI* gene in *P. erlangeri* and *T. kachowskii* revealed that haplotypes do not group on the phylogenies according to geography. Instead, eastern and western samples are interspersed over the phylogenies (Fig. 2). This is confirmed by the AMOVA analyses based on mitochondrial sequences which indicated that most of the variation is found within populations and that among group variation does not contribute to the overall genetic variance between populations east and west of the GRV (Table 2). In *P. erlangeri*, two deeply divergent lineages were recovered in 3 individuals from the forests of the southwest. These two lineages differ on average from the main clade by 10.8 and 3.3 %, respectively (Fig. 2). This amount of divergence is unusual within species, yet the persistence of ancestral mitochondrial lineages in frog populations has been observed in other taxa [23–25]. It should be noted that the frogs carrying these divergent lineages come from localities where the main clade was recovered and that they are undistinguishable from other *P. erlangeri* with respect to morphology or nuclear variation.

In *P. erlangeri* and *T. kachowskii*, all the nuclear genes revealed the same patterns, i.e. extensive allele sharing between populations on the east and the west side of the GRV, and the level of differentiation between east and west estimated by *Fst* were low and non-significant at all loci (Table 3). The *Structure* analysis determined that the most likely number of populations in these two species was 1 based on the Pr(K|X) criterion and as 2 by the delta-K criterion, which is not designed to chose the best value of K when K < 2 (Table 4). Similarly, the *Structurama* analysis provides strong support in favor of a single population (Table 4). Finally the AMOVA analyses based on the nuclear datasets indicate that the vast majority of the variation is found within population (~92 and 99 % in *P. erlangeri* and *T. kachowskii*, respectively) (Table 2). Clearly, all the methods used to detect genetic structure in *P. erlangeri* and *T. kachowskii*, using either the mitochondrial or the nuclear datasets, demonstrate that these species consist of a single gene pool and that the GRV has no impact on their genetic structure.

In *P.* cf. *neumanni 1*, the only species limited to the western side of the GRV, we also failed to find any population structure. The mitochondrial phylogeny did not reveal any geographic grouping (not shown) and the *Structurama* and *Structure* analyses strongly supported the presence of a single gene pool (Table 4). In addition, *F*_*st*_ calculated between all pairs of population were consistently lower than 0.1 confirming the lack of genetic structure in this species (data not shown).

The estimates of population size calculated with BPP are highly consistent across priors and are similar among taxa, from ~400,000 in *P*. cf. *neumanni 1* and *T. kachowskii* to ~600,000 in *P. erlangeri*. The demographic trajectory constructed with EBSP looks similar among the three low elevation species and suggests a progressive 10-fold population expansion beginning ~400,000 years ago, identical to the expansion observed in *L. gramineus* (Fig. 4). A past population expansion is also supported by the fact that almost all values of Tajima’s D and Fu’s F are negative for the mitochondrial and nuclear genes (Table 3).

## Discussion

We reconstructed the evolutionary history of seven frog species with different elevational and ecological preferences and we examined the impact the GRV has had on their population structure. Our results can be summarized as follow: (1) In species that are found at high elevations (*P*. cf. *neumanni* 2, *P. cooperi*, *Amietia* sp. and *L. gramineus*), populations east and west of the GRV are genetically distinct; (2) In species that are found at elevations under 2500 m (*P. erlangeri* and *T. kachowskii*) there is no genetic differentiation between populations on each side of the GRV; (3) Genetic variation differs considerably among Ethiopian frog species suggesting differences in effective population size and demographic history; (4) Several species (*L. gramineus*, *P. erlangeri*, *P*. cf. *neumanni* 1, *T. kachowskii*) exhibit the signature of a gradual demographic expansion starting ~400,000 years ago.

Before further discussing our results, a word of caution is necessary. The number of genes analyzed here is relatively small and the amount of variation at nuclear protein coding genes is low, particularly in *Amietia* sp*.* and *P. cooperi*. In addition, there is some uncertainty about mutation rates in frogs, particularly for nuclear genes. Thus, estimates of divergence time or effective population size need to be interpreted with caution. With these caveats in mind, it remains that our most significant results do not rely on mutation rates. The mitochondrial phylogeny, the *Structure* and *Structurama* analyses and the AMOVA all support a role of the GRV in the splitting of the four highland species into two gene pools. Similarly, the mitochondrial and nuclear datasets are congruent in showing a lack of structure in species distributed at elevations lower than 2500 m. It is unlikely that more data would contradict these conclusions, although additional analyses could yield more accurate estimates of the timing of population subdivision and the effective population size of our focal species. In addition, it should be acceptable to compare variation and relative divergence among species since the nature of the datasets used here are comparable across species, comprising 1 protein-coding mitochondrial gene and a combination of 3 to 5 nuclear genes.

Our analysis emphasizes the role of the GRV as a biogeographic barrier for highland adapted species. We demonstrated that the GRV has prevented gene flow between eastern and western populations for the last ~0.4 my in *Amietia* sp*.*, *P*. cf. *neumanni* 2 and *P. cooperi* and for the last 2.0 my in *L. gramineus*. The absence of recent gene flow can easily be explained by the current climatic conditions in the floor of the GRV that are not favorable to any of the highland species (Fig. 2). However, the population divergences we dated in the early to mid-Pleistocene suggest that the climatic oscillations of the last 0.5 my did not result in any detectable gene flow between the east and the west of the GRV. Ecological niche models for the LGM indicate that climatic conditions in the GRV were not favorable for some species (*P*. cf. *neumanni* 2) but that they could have been for others (*Amietia* sp., *P. cooperi*, *L. gramineus*), yet did not result in substantial migration (Fig. 1). Thus, climate alone does not fully explain the persistence of genetic differentiation in highland species. Another factor that could have played a role is the topography of the GRV, in particular the steep slopes in its upper portions. The margins of the Ethiopian GRV are comparable to an 800 m wall (from 1500 m asl in the floor of the GRV to 2300 m asl on the plateau) and are likely to constitute insurmountable barriers to dispersal for frogs. Recent studies showed that mountain ridges and deep slopes can act as a dispersal barrier for amphibians [26–28], because of physiological limitations [28] or because of the high energy cost of climbing steep slopes [26]. Indeed, an effect of topography has been demonstrated in a number of frog species, including *Rana luteiventris* [26, 29], *Epipedobates femoralis* [28], and *Atelopus varius* [30]. It is highly likely that a combination of climatic and topographical factors is responsible for the pattern of divergence we report for Ethiopian taxa, although it is not possible to decipher between these two factors with our data.

It remains, however, that *P. cooperi*, *Amietia* sp*.*, *L. gramineus* and *P.* cf. *neumanni 2* are present on both side of the GRV and that at some point the GRV could be crossed by these species. One possibility is that in the past the floor of the GRV was higher relative to the plateaus. It is well known that elevation of the GRV has changed through time [31] and it is plausible that the gradual down-faulting of the GRV’s floor resulted in the formation of a vicariant barrier, splitting the distribution of these species in two. An alternative possibility is that the conditions in Ethiopia were sufficiently cool and humid until ~0.4 my ago and that the distribution of the species were then continuous. It is only with the increased aridification of Eastern Africa, particularly in the last half million year, that the GRV became a true barrier to dispersal [6].

It appears that the split between populations east and west of the GRV is not concomitant in all species. The divergence time between east and west are similar between *Amietia* sp*., P*. cf. *neumanni* 2 and *P. cooperi* at ~0.4 my but the east-west split is substantially older in *L. gramineu*s (at ~2.1 my) as in *Xenopus clivii* and *X. largeni* (at ~1.0 to 3.5 my) [16]. These differences could be due to different dispersal abilities or to different tolerance to dry conditions. *Amietia* sp*., P. cooperi*, and *P.* cf. *neumanni* 2 are relatively agile frogs which could easily colonize new favorable habitats whereas *L. gramineus* is a slow moving species which requires the persistence of moisture in soils to accommodate its fossorial ecology. Frogs of the genus *Xenopus* are exclusively aquatic and it is unlikely they are capable of long distance migrations, although they are capable of short distance dispersal on land. These differences in dispersal abilities are particularly relevant in the context of the GRV since the topography of the margin of the rift could constitute a considerably more difficult barrier to dispersal for fossorial and aquatic species.

Our study of *Amietia* sp*.* indicates that the Blue Nile Valley is acting as a biogeographic barrier. The ENMs clearly show that the climatic conditions in the valley are currently not favorable to this species but they also reveal that this was true during the Last Glacial Maximum. The combination of extremely steep slopes (the canyon is 1400 m deep) with the persistent dry conditions on the valley floor is likely to prevent dispersal of highland frogs. Such a role of the Blue Nile valley had previously been detected in *Xenopus clivii* and is supported by the presence of endemic species restricted north of the Blue Nile (e.g. *P.* cf. *neumanni* 5, *Leptopelis yaldeni*) and by the absence of several species that are found immediately south of the valley (*P. cooperi*, *P*. cf. *neumanni* 1, *L. gramineus*).

The amount of genetic variation differs considerably among species, reflecting differences in effective population size (N_e_) and demographic history. The N_e_ of the species found at low elevation (*T. kachowskii*, *P.* cf. *neumanni* 1 and *P. erlangeri*) are relatively similar (~400,000 to 700,000 individuals) and substantially higher than the N_e_ of the highland species (*Amietia* sp*.*, *P. cooperi* and *P*. cf. *neumanni* 2) which are 4 to 10 times lower (~40,000 to ~120,000). This difference is not due to a recent bottleneck in highland species since none of the values of Tajima’s D and Fu’s F are significantly positive and the EBSP either showed a recent demographic expansion (*P.* cf. *neumanni* 2; Fig. 4) or demographic stability (*Amietia* sp., *P. cooperi*; analysis not shown). Instead, these differences are best explained by the large and gradual demographic expansion of lowland species starting ~400,000 year ago. This demographic trajectory is consistent with the progressive warming of East-Africa in the Pleistocene [6], which could have resulted in the expansion of habitats favorable to lowland species (for example savanna for *T. kachowskii*) as well as causing the fragmentation of tropical forests, thus generating novel habitats to species living in clearings or in forest margins (*P.* cf. *neumanni* 1 and *P. erlangeri*). However, it is difficult to evaluate the validity of this scenario given the number of uncertainties about the geological and climatic history of this region.

*Leptopelis gramineus* is the exception to the trend described above, its highland population (Arsi) being large (~1,000,000) and having experienced a 10-fold demographic expansion, comparable to the one observed in lowland species. The genus *Leptopelis* is a typical inhabitant of African tropical forests and the high elevation grasslands are not typical habitats. Thus it is likely that the tropical rain forests constitute the ancestral habitat of *L. gramineus* (as suggested by preliminary phylogenetic analyses of the genus; [32] and Reyes-Velasco et al., unpublished data) and that a niche shift occurred concomitantly or posteriorly to the split between the Arsi population and the forests populations (Kasha, Kibre Mengist), dated at ~1 my. We propose that the ecological shift from a forest to a highland habitat could have resulted in the colonization of an empty ecological niche on the plateaus and in a large demographic expansion. This hypothesis is highly speculative at this point and will require validation by additional research.

## Conclusions

We found substantial genetic differentiation in highland species, demonstrating a role of the GRV as a biogeographic barrier, but an absence of structure in species found below 2500 m. The absence of gene flow between highland populations for the last 0.4 my, together with the demographic expansion of low elevation species starting around the same time, suggests a major change in the environmental conditions in Ethiopia in the last half million year and is consistent with the progressive aridification of Eastern Africa during the Pleistocene.

## Methods

### Sample collection

Samples were collected in 101 different localities on both sides of the GRV (Fig. 1). Collecting took place during the rainy season in July and August 2011 and from June to August 2013. Specimens of *Tomopterna kachowskii* (N = 40), *Amietia* sp*.* (N = 35), *Leptopelis gramineus* (N = 34), *Ptychadena cooperi* (N = 31), *P. erlangeri* (N = 36), *P*. cf. *neumanni* 1 (N = 49) and *P.* cf.*neumanni* 2 (N = 60) were collected from 16, 13, 17, 14, 12, 15 and 14 localities, respectively. For all species, except *P.* cf. *neumanni* 1, samples were collected on both sides of the GRV. Collection and export permits were obtained from the Ethiopian Wildlife Conservation Agency. The origin of each sample is listed in Additional file 1: Table S1. Dissections were performed immediately after the frogs were euthanized by ventral application of benzocaine [33]. Liver or muscle samples were preserved in 90 % ethanol. All specimens were deposited at the Natural History Museum of Addis Ababa University.

### Molecular analyses

Genomic DNA was isolated from the ethanol-preserved tissues using the Wizard SV® genomic DNA purification system (Promega). Two mitochondrial genes were amplified and sequenced: the cytochrome oxidase 1 gene (*COI*) (a 505 bp fragment in *T. kachowskii* and *L. gramineus*, and a 447 bp fragment in all *Ptychadena*) and 962 bp of the NADH dehydrogenase subunit II (*ND2*) in *Amietia* sp*.* Depending on the species, 3 to 5 nuclear genes were amplified and sequenced. In *T. kachowskii*, a region of 614 bp in the recombinase activating gene 1 (*Rag-1*), a region of 791 bp in the second exon of the sodium/calcium exchanger gene 1 (*NCX1*), a 452 bp fragment in the brain-derived neurotrophic factor gene (*BDNF*) and 785 bp of the solute carrier family 8 member 3 gene (*SLC8A3*). In *Amietia* sp*.*, we sequenced 791 bp of *Rag-1*, 563 bp of *BDNF* and a 540 bp region in the first exon of the tyrosinase gene (*Tyr*). In *L. gramineus* we sequenced 699 bp of *Rag-1*, 554 bp of *NCX1*, 400 bp of *BDNF*, 442 bp of *Tyr* and a 566 bp region in the recombinase activating gene 2 (*Rag-2*). For *Ptychadena*, the same four nuclear genes analyzed in [17] were used, namely 523 bp of *Rag-1*, 668 bp of *NCX1*, 331 bp of *Tyr* and 447 bp in the second exon of the chemokine receptor type 4 gene (*CXCR4*).

PCR conditions consisted of an initial denaturation step at 94C for 2 min, followed by 30 cycles for 30s at 94C, 30s at 48–60C depending on the primer pairs, 1 min at 72C, then a final extension at 72C for 1 min. The sequences of the primers and the annealing temperatures are presented in Additional file 2: Table S2. PCR products were purified and sequenced in both directions by the High Throughput Genomics Unit at the University of Washington in Seattle. Chromatograms were imported into Geneious Pro version 6.4 created by Biomatters available at http://www.geneious.com. Putative heterozygotes were assessed based on quality score and verified by visual inspection of the chromatograms. The reverse and forward reads were assembled into contigs and alignments were generated for each locus. The gametic phase of each nuclear haplotype was resolved using PHASE 2.1 implemented in DnaSP version 5.

### Phylogenetic analyses

Mitochondrial genes were analyzed phylogenetically using maximum-likelihood and Bayesian methods. The substitution model that best fits the data was determined using the Bayesian Information Criterion in MEGA 5.0 [34]. Maximum likelihood phylogenies were reconstructed using the MEGA 5.0 and the robustness of the nodes was assessed using 1000 bootstrap replicates. Bayesian phylogenies were reconstructed using MrBayes 3.2 [35]. Analyses were run for 20,000,000 generations and we sampled 10,000 trees, discarding the first 1000 as burn-in. The time to most recent common ancestor (TMRCA) for each mitochondrial lineages and the divergence between mitochondrial lineages were estimated using the program BEAST v1.8 [36]. Analyses were performed using the HKY + Gamma model of substitution. We used a coalescent tree prior, an uncorrelated relaxed clock and we assumed a constant population size. Analyses were ran for 100,000,000 generations from which 100,000 trees were sampled following a 10 % burn-in. Results were checked for convergence using Tracer v1.6 [37]. Since *COI* and *ND2* evolve at relatively similar rates in anurans [17], we estimated divergence time using the ND2 rate of 0.957 % per lineage per million years [38].

### Population structure

The population structure within each species was inferred using several approaches. First, we used the Bayesian clustering program *Structure v2.3.3.* [39] on the nuclear data. *Structure* estimates the likelihood of a user-set number of clusters (*K*) and assigns individuals to each cluster. We ran the model with or without admixture for 200,000 generations, with 10,000 discarded as burn-in. Five runs were performed for each value of *K* ranging from 1 to 6. We assessed the level of support for each value of *K* using the ad hoc approach proposed by Pritchard et al. [39] as well as the delta-*K* criterion of Evanno et al. [40], calculated with *Structure Harvester* [41]. We also used a second Bayesian clustering approach, implemented in *Structurama* version 2.0 [42]. *Structurama* treats the number of population as a random variable using a Dirichlet prior and assigns individuals to each inferred populations. We performed the analysis using five priors with a mean number of clusters 1 to 6. We also treated the concentration parameter as an hyper-parameter and used a gamma hyper-prior G(2.5, 0.5). The Markov Chain Monte Carlo was run for 1,000,000 cycles, sampled every 100th cycle and discarded the first 5000 as burn-in. We used the “no admixture” model on all analyses. Relying solely on Bayesian clustering analyses to infer the number of populations in a sample has been questioned [43–45]. Thus, we also assessed the level of differentiation between each sampled populations by calculating *F*_*st*_ for each gene and for the entire nuclear dataset. Individuals sampled 20 km from each others were pooled and considered part of the same population. These calculations were performed using Arlequin v3.5 [46]. To assess the effect of the Rift Valley on population structure we performed an analysis of molecular variance (AMOVA) [47] on the mitochondrial datasets and on a concatenated nuclear dataset using Arlequin [46]. Populations were divided into two groups, the populations west of the Rift and the populations east of the Rift. The AMOVA partitions hierarchically genetic variation among populations relative to the total sample, among populations within regions, and among regions. Significance of the results was assessed by 10,000 permutations of the data matrix. For each population (defined by Bayesian clustering or by geography), we calculated a number of descriptive statistics including the number of segregating site (S), the number of haplotypes (h), the nucleotide diversity (π), and the Waterson’s estimator of nucleotide diversity (θ). Neutrality or demographic changes was assessed using Tajima’s D [48] and Fu’s F [49]. These calculations were performed using the program DnaSP version 5 [50].

### Timing of divergence and demographic history

We estimated the divergence time between populations using the species tree approach implemented in *BEAST [51]. *BEAST implements a probabilistic framework using sequences from several unlinked loci and several individuals per populations to infer a species/population tree and to estimate the divergence time of the populations. *BEAST analyses were performed using the mitochondrial genes and 3 to 5 nuclear genes, depending on the species. The substitution models used were HKY + Gamma for the mitochondrial genes and HKY for the nuclear genes. For divergence time estimation we used the mitochondrial mutation rate of 0.957 %. We placed normally distributed prior probabilities around the mutation parameters for each nuclear gene with a starting mean of 1. The divergence times were estimated under the uncorrelated relaxed-clock tree model with a Yule prior. Analyses were run twice for 400,000,000 generations, sampling every 10,000 generations for a total of 40,000 trees. Convergence between the runs was monitored using the effective sample size (ESS) values obtained using Tracer v1.6. The initial 10 % of the runs were discarded as burn-in and maximum-clade credibility trees with divergence time estimates were obtained using TreeAnnotator in BEAST v1.8 [36].

Divergence times were estimated using a second method, implemented in G-PHoCS [52]. Using a coalescent framework, G-PHoCS estimates key population genetics parameters including population sizes (θ), population divergence times (τ) and migration rates (m) from unlinked nuclear loci. G-PHoCS integrates all possible phases of diploid genotypic data, thus removing a potential source of error in phylogeographic analyses. We evaluated the effect of different priors of the gamma distribution G(α,β) for the θ and τ parameters, which were ~ G(2,10), ~G(2,1000) and ~ G(2,2000). For each set of priors we ran five replicates with different seeds for 500,000 generations, with a sampling interval of 50 generations and we assessed convergence of separate runs using Tracer v1.6. Demographic parameters estimated by G-PHoCS are given as relative values as they are scaled by the mutation rate, which requires a conversion to obtain values in time or number of individuals [52, 53]. As we do not have reliable outgroups with accurate divergence time for any of the taxa analyzed here, we used an average of the mutation rates calculated in [17] for 4 nuclear genes of 0.065 % per my, which is consistent with the 16-fold difference between the mitochondrial and nuclear mutation rate in anurans estimated by Crawford [38]. Unfortunately there is no information available on the reproduction of these species, particularly in terms of generation time. Considering that the activity (and consequently the growth) of these species is probably limited during the dry season, it is plausible to assume an age at maturity of 2 years. As G-PHoCS is designed to analyze multiple populations, we estimated the effective population size of *T. kachowskii*, *P. erlangeri* and *P*. cf. *neumanni 1* (that do not show any population structure; see results) using the BPP software [54], which uses the same coalescent framework as G-PHoCS.

The demographic trajectory of each population was further analyzed using the extended Bayesian skyline plots (EBSP) on the multi-locus data [55]. The EBSP uses the coalescent history of multiple genomic loci to estimate the number and extent of population size changes in the past. For the EBSP, all loci were assigned different substitution models, clock models and trees. The clock rates for the nuclear genes were assigned a uniform [0,1] prior distribution and the rates were estimated relative to the mitochondrial sequences.

### Ecological niche modeling

Habitat suitability maps for the present time and for the LGM (21ky) were obtained by means of ecological niche models (ENMs; [22]) where we used the presence data obtained from our own observations. The presence data include all the individuals analyzed here, as well as additional collecting points not included in the present study. ENMs were built by means of MaxEnt [56] using default parameters and settings, and with Worldclim bioclimatic variables [57] as environmental predictors. We used an uncorrelated (r < 0.7) dataset of six bioclimatic variables out of the 19 variables available from WorldClim. The reduction in the number of variables was done in order to reduce variable collinearity and model overfitting, which have been indicated to negatively affect model predictions, particularly when projecting across space or time [58, 59]. These variables were mean diurnal range (Bio2), isothermality (Bio3), mean temperature of warmest quarter (Bio10), precipitation of wettest month (Bio13), precipitation of warmest quarter (Bio18), and precipitation of coldest quarter (Bio19). As extent for training the model we used our sampling area, as defined by the maximum convex polygons of all species’ locations, plus a buffer area of 1°. The final model for the present time is the average prediction of 50 model replicates. On each replicate, the occurrence data is split in a training set (80 %) and a test set (20 %). The number of occurrences per species ranged from 13 to 24 (Table 1). ENMs parameterized for the present time were projected into LGM conditions (21ky ago) obtained from the WorldClim database. LGM was represented by three different climatic models: the Community Climate System Model (CCSM4), the Model for Interdisciplinary Research on Climate – Earth System Model (MIROC-ESM) and the Max Plank Institute – Earth System Model (MPI-ESM). Using three different climate models allows us to account for uncertainty associated to LGM climate data. Continuous models for both the present and the LGM times were turned into binary distribution ranges using the threshold value that maximizes the sum of the sensitivity and specificity of the model in the test data sets [60]. In order to address the issue of non-analogous climates [61], we used the clamped projections maps provided by MaxEnt, where environmental layers and projections are truncated to maximum and minimum environmental and projected values in the training data set. Additionally, MaxEnt also yield clamping maps that inform for each simulation and for each cell the absolute difference between prediction values with and without clamping. In our final projected maps we only project the model to those areas where this difference is < 0.1, thus ensuring that uncertainty due to non-analogous climates is low.

## Additional files

Additional file 1: Table S1. Samples used in this study, including collecting localities and GenBank accession numbers. (XLSX 51 kb)
Additional file 2: Table S2. Primers used in this study. (XLSX 11 kb)

## Abbreviations

asl: Above sea level
ENM: Ecological niche model
GRV: Great Rift Valley
LGM: Last Glacial Maximum
my: Million year
